# Seroprevalence and risk factor analysis for exposure to equine encephalosis virus in Israel, Palestine and Jordan

**DOI:** 10.1002/vms3.58

**Published:** 2017-03-06

**Authors:** Sharon Tirosh‐Levy, Boris Gelman, Doni Zivotofsky, Lara Quraan, Evgeny Khinich, Abdelmajeed Nasereddin, Ziad Abdeen, Amir Steinman

**Affiliations:** ^1^ Koret School of Veterinary Medicine The Robert H. Smith Faculty of Agriculture Food and Environment The Hebrew University of Jerusalem Rehovot Israel; ^2^ Virology Division Kimron Veterinary Institute Beit‐Dagan Israel; ^3^ Vet Practice Mechalkey Hamayim 1 Jerusalem Israel; ^4^ Al‐Quds Public Health Society Jerusalem Palestine and Al‐Quds Nutrition and Health Research Institute Faculty of Medicine Al‐Quds University Abu‐Deis Palestine

**Keywords:** Equine encephalosis virus, seroprevalence, arbovirus, horse

## Abstract

Equine encephalosis virus (EEV) is an orbivirus transmitted by *Culicoides* species. Most infected horses show mild clinical signs and mortality is usually very low. EEV is closely related and similarly transmitted to other, more pathogenic and economically important, orbiviruses such as African horse sickness virus (AHSV), bluetongue virus (BTV) and epizootic haemorrhagic disease viruses (EHDV), and may serve as an indicator for possible transmission of the latter. Israel has been reported to be endemic for EEV since 2001. This study was initiated to re‐evaluate the current seroprevalence and risk factors for EEV exposure in Israel, and to assess, for the first time, the seroprevalence of EEV in Palestine and Jordan. Three hundred and sixteen serum samples were collected from apparently healthy horses at 21 farms in Israel, 66 horses at nine farms in Palestine and 100 horses at three farms in Jordan. The presence of EEV antibodies was detected by a serum neutralization assay. Seroprevalence of EEV was 58.2% (184/316 horses) in Israel, 48.5% (32/66 horses) in Palestine and 2% (2/100 horses) in Jordan. Seroprevalence in Jordan was significantly lower than in Israel and Palestine (*P* < 0.001). The farm (*P* < 0.001) and horse age (*P* = 0.003) were found as significant risk factors for EEV exposure in Israel in multivariable statistical analysis. The results of this study further demonstrate that EEV is no longer limited to South Africa and is endemic in both Israel and Palestine and horses in Jordan were also exposed to this virus emphasizing the potential of pathogens to invade new ecological niches.

## Introduction

Equine encephalosis virus (EEV) is an arthropod‐borne virus of equids that belongs to the genus Orbivirus and the family Reoviridae, and is transmitted by several midge (*Culicoides)* species. Most infected horses show either no obvious signs or develop very mild clinical signs, which may include fever, inappetence, congestion and mild icterus of the mucous membranes. Severe signs are rare and mortality is very low (Hinchcliff 2007). EEV is closely related to other orbiviruses including African horse sickness virus (AHSV), which can cause a highly fatal disease in horses; bluetongue virus (BTV) and epizootic haemorrhagic disease virus (EHDV), which both have significant veterinary and economic effects on ruminants (Attoui & Jaafar 2015). Since all the mentioned orbiviruses are transmitted by *Culicoides* biting midges, many similar environmental parameters may influence their prevalence and distribution.

Prior to 2008 EEV was only known to be endemic in South Africa, with seven serotypes circulating in the area (Howell *et al*. 2002, 2008). Since then it was reported in several locations in East and West Africa, as well as in Israel (Mildenberg *et al*. 2009; Oura *et al*. 2012; Attoui & Jaafar 2015). Retrospective studies from Israel demonstrated that this virus was present in the horse population from at least 2001 (Wescott *et al*. 2013), and that the prevalent strain in Israel was more similar to a strain isolated in Gambia than to the South African strains (Howell *et al*. 2002; Aharonson‐Raz *et al*. 2011; Oura *et al*. 2012). It was also demonstrated that EEV seroprevalence and rate of infection varied at different time points, and was influenced mainly by herd immunity and weather conditions (Aharonson‐Raz *et al*. 2015). Israel's unique location at the junction of three continents, Europe, Asia and Africa, makes this region particularly interesting in studying the spread of infectious diseases. Palestine and Jordan are located on the Eastern border of Israel. There is a large movement of horses between Palestine and Israel for different purposes, such as trade, sport and for medical treatment and transfer of small number of horses from Jordan for medical treatment in Israel. This study was initiated to re‐evaluate the current seroprevalence and risk factors for EEV exposure in Israel, and to assess, for the first time, its seroprevalence in Palestine and Jordan. Serum samples were collected from horses at different farms in Israel, Palestine and Jordan and the presence of EEV antibodies was detected by a serum neutralization assay.

## Materials and Methods

### Equine serum samples

Three hundred and sixteen serum samples were collected from apparently healthy horses at 21 farms in Israel during February‐March 2015. At each farm all available horses were sampled, (4–38 horses). The Israeli equine population is estimated to be approximately 30 000 horses, but detailed information of horses and farms is lacking. A sample size of 270–326 horses was calculated for estimated prevalence of 77%, as was reported in 2013. Farms were selected to represent the estimated geographical distribution of the equine population in Israel, with more horses in the north and centre of Israel and fewer horses in the south of Israel. Farms were also selected to reflect different management regimes commonly used in different geographical regions, with both pastured and stalled horses in the north, and mostly stalled horses in the centre.

None of the sampled horses was imported from another country or travelled to Palestine. Horses were used for showing, pleasure riding or for sport, therefore some of the horses did travel in and out of the premises inside Israel. Data for each horse were collected from farm managers and included sex, breed, age, housing and recent health condition. Rectal temperature was measured during sampling. None of the horses had a history of recent febrile disease and all had rectal temperature within normal limits (36.5–38.5°C). Blood was collected from the jugular vein of each horse into a sterile vacuum tube without an anticoagulant agent. Sera were obtained from clotted blood samples by centrifugation (3000*g* for 8 min) and stored at −80°C until usage.

Serum was collected and stored in a similar fashion from 66 apparently healthy horses at nine farms in Palestine during July‐September 2014, between two and 30 horses at each farm. The sampled farms in Palestine did not necessarily reflect the distribution of farms, since this is not known, however, samples were collected from different regions of Palestine. None of the sampled horses was imported from another country or left Palestine. Serum was also collected from 100 horses at three farms in Jordan, all from a small geographical area near Amman, during February 2015, between 10 and 50 horses at each farm. None of the horses were imported from another country within the year prior to sampling. Horse details including sex, breed and age were collected from farm managers. None of the horses had signs or a recent history of febrile disease and none had travelled to Israel in the past.

Blood collections were performed with the owners' consent, and the study was approved by the Internal Ethics Review Committee of the Koret School of Veterinary Medicine, The Hebrew University.

### Serum neutralization test for the detection of EEV antibodies

The Israeli EEV strains (H3, H8) (Aharonson‐Raz *et al*. 2011) were used as the neutralized viruses for the serum neutralization test. These viruses were isolated on Vero cells in 2009 during the EEV outbreak in Israel, and formed a distinct group, similar to EEV‐3, but with characteristics of both south‐African serologic groups (Aharonson‐Raz *et al*. 2011). The serum neutralization test was performed as described previously (Aharonson‐Raz *et al*. 2015). A result was considered positive if cell cytopathic effects were prevented, starting from a dilution of 1:4 (Aharonson‐Raz *et al*. 2015).

### Statistical analysis

Seroprevalence was estimated separately for each of the study populations (Israel, Palestine and Jordan). Statistical significance of the differences in the prevalence between these populations was assessed by the two‐sided *χ* ^2^ test. Risk factors associated with exposure to EEV were assessed for each population separately. Association with nominal independent variables was assessed using the *χ* ^2^ test, and odds ratios were calculated. Association with quantitative parameters was assessed using the t‐test. Association between variables was considered statistically significant when the *P*‐value was less than 0.05. All significant parameters in the univariable analysis were included in a multivariable analysis, using a forward‐stepwise model. The data for the Israeli and Palestinian populations were also analysed using generalized estimating equation with a logit link function, with the farm set as a subject (i.e. random variable) and with an exchangeable working correlation matrix. The analysis was performed, using SPSS 22.0^®^ and Win Pepi 11.43^®^ statistical software.

## Results

### Study population

**Figure 1:**
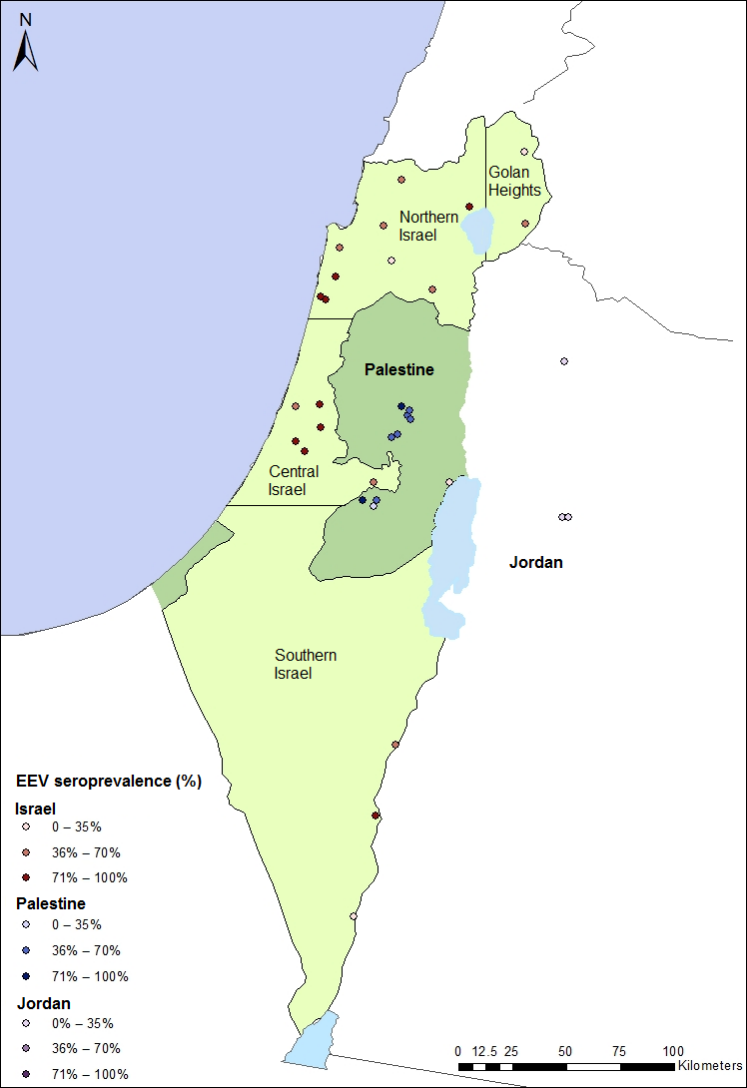
Geographical distribution of the farms sampled at the study and EEV seroprevalence at each farm. Colour gradient correlates with EEV seroprevalence in each location, with darker colours represent higher seroprevalence.

The horse population sampled in Israel comprised of 316 horses from 21 farms (Fig. 1). The farms were selected to represent the distribution of the horse population in Israel, with higher density in the north and centre of Israel, and fewer horses in the south. One hundred and forty‐two horses were sampled in the north (44.9%), 105 in the centre (33.2%), 33 in the south (10.4%) and 36 in the Golan Heights (11.4%) (in Fig. 1. Israel was divided into four regions). Most of the horses were mixed bred (196 horses, 62%) and the rest were of different breeds, including Quarter horses (60 horses, 19%), Ponies (11 horses, 3.5%), Tennessee Walking horses (10 horses, 3.2%), Paint horses (9 horses, 2.8%), Warmbloods (7 horses, 2.2%), Arabians (6 horses, 1.9%), Appaloosas (4 horses, 1.3%), Missouri Foxtrots (3 horses, 0.9%), 2 Thoroughbreds (0.6%), 2 Tinkers (0.6%) and one Shire (0.3%). The population distributed almost equally between male and female horses, with 149 mares (47.2%), 156 Geldings (49.4%) and 11 stallions (3.5%). The mean age was 10.8 (±5.4) years, ages ranged between 9 months and 30 years. Many of the farms located in the north and Golan Heights kept the horses in pasture (117 horses, 37%), the rest of the horses were housed either in stalls (118 horses, 37.3%) of paddocks (81 horses, 25.6%).

In Palestine, 66 horses were sampled at nine farms, with between 3 and 30 horses in each farm (Fig. 1). The horses were of different breeds, including Quarter horses (20 horses, 30.3%), Arabians (12 horses, 18.2%), Thoroughbreds (six horses, 9.1%), Paint horses (five horses, 7.6%), Appaloosas (three horses, 4.5%), Ponies (two horses, 3%), one Warmblood (1.5%) and the rest were mixed bred horses (17 horses, 25.8%). The population distributed almost equally between male and female horses, with 32 mares (48.5%), 19 geldings (28.8%) and 15 stallions (22.7%). Ages ranged between 6 months and 20 years, with a mean of 7.8 (±4.7) years. Information regarding the management and housing was not available.

In Jordan 100 horses were sampled at three farms, between 10 and 50 horses in each farm. Unfortunately, the demographic data of the horses sampled were unavailable.

### Seroprevalence of EEV in Israel, Palestine and Jordan

**Figure 2:**
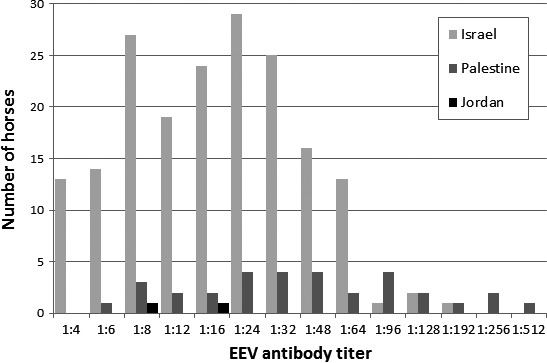
The distribution of EEV‐specific antibody titres, as detected by serum neutralization assay, in asymptomatic horses in Israel (light grey), the Palestine (dark grey) and Jordan (black).

Of the 316 horses sampled in Israel 184 were seropositive for EEV (58.2%, 95% CI: 52.58–63.72). In Palestine 32 of 66 horses were seropositive (48.5%, 95% CI: 35.99–61.12), and in Jordan only two of 100 horses were seropositive (2%, 95% CI: 0.2–7) (Fig. 1). Seroprevalence in Jordan was significantly lower than in Israel and Palestine (*P* < 0.001), and horses in Israel were 68 times more likely to be exposed to EEV than horses in Jordan (odds ratio (OR): 68.3, 95% CI: 16.66–279.9). The seroprevalence in Israel and Palestine did not differ statistically (*P* = 0.172). The distribution of titre values differed between Israel, Palestine and Jordan. The two seropositive horses in Jordan had relatively low antibody titres, whereas in Israel and Palestine individual antibody titres varied (Fig. 2).

### Risk factors for exposure to EEV in Israel and Palestine

Since EEV seroprevalence did not differ significantly in Israel and Palestine, risk factors for exposure were analysed in these two populations together. Factors that were found to be significantly associated with exposure to EEV in univariable analysis were the farm (*P* < 0.001), geographical area (*P* < 0.001), horse breed (*P* = 0.027), horse sex (*P* = 0.01), housing (*P* = 0.003) and age (*P* < 0.001) (Table 1).

**Table 1:** Univariale analysis of risk factors considered for an association with Equine enchephalosis virus seropositivity in horses sampled in Israel and Palestine

Variable	Category (n)	Number of carriers (%)	OR (95% CI)	*P*‐value (fisher's two tailed)	*P*‐value (*χ*2)
	Israel	184/316 (58.2%)	1.48 (0.87–2.51)	0.172	0.172
	Palestine	32/66 (48.5%)	Ref		
**Area**	Northern Israel	78/142 (54.9%)	3.66 (1.62–8.25)	**<0.001**	**<0.001**
	Central Israel	85/105 (81%)	12.75 (5.25–30.98)	**<0.001**	
	Southern Israel	12/33 (36.4%)	1.71 (0.62–4.76)	0.433	
	Golan Heights	9/36 (25%)	Ref		
**Sex**	Mare	92/181 (50.8%)	0.57 (0.37–0.87)	**0.01**	**0.01**
	Stallion	11/26 (42.3%)	0.4 (0.18–0.92)	**0.05**	
	Gelding	113/175 (64.6%)	Ref		
**Age**	Under three years	3/27 (11.1%)	Ref		**<0.001**
	Three to six years	34/70 (48.6%)	7.56 (2.13–26.81)	**<0.001**	
	Six to nine years	50/84 (59.5%)	11.76 (3.35–4126)	**<0.001**	
	Nine to twelve years	50/83 (60.2%)	12.12 (3.45–42.57)	**<0.001**	
	Over twelve years	74/111 (66.7%)	16 (4.62–55.38)	**<0.001**	** **
**Breed**	Mixed	114/213 (53.5%)	Ref		**0.027**
	Thouroghbred	6/8 (75%)	2.61 (0.57–11.92)	0.295	
	Missurie Foxtrot	3/3 (100%)	6.08 (0.46–80.29)	0.252	
	Paint Horse	5/14 (35.7%)	0.48 (0.16–1.43)	0.27	
	Quarter Horse	48/80 (60%)	1.3 (0.77–2.19)	0.357	
	Shire	1/1 (100%)	–	1	
	Warmblood	6/8 (75%)	2.61 (0.57–11.92)	0.295	
	Pony	10/13 (76.9%)	2.89 (0.81–10.29)	0.15	
	Tinker	0/2 (0%)	–	0.22	
	Tennessee Walking Horse	9/10 (90%)	7.82 (1.08–56.51)	**0.025**	
	Appaloosa	3/7 (42.9%)	0.65 (0.16–.67)	0.708	
	Arabian	7/18 (38.9%)	0.55 (0.21–1.44)	0.32	
**Housing**	Stall	83/118 (70.3%)	2.41 (1.41–4.11)	**>0.001**	**0.003**
	Paddock	43/81 (53.1%)	1.15 (0.65–2.02)	0.666	
	Pasture	58/117 (49.6%)	Ref		
**Colour**	Dark	99/167 (59.3%)	0.98 (0.54–1.81)	1	0.781
	Light	50/91 (54.9%)	0.82 (0.42–1.61)	0.612	
	Coloured	34/57 (59.6%)	Ref		

Statistically significant values are written in bold

**Figure 3:**
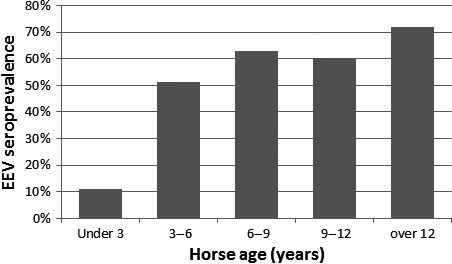
The association of EEV seroprevalence with age. No significant differences were observed between the different groups, except for the youngest age group (under 3 years) in which the seroprevalence was significantly lower (*P* = 0.005). A linear correlation was found between age and seroprevalence (*P* < 0.001).

Seroprevalence in different farms ranged between 14% and 100%. Farms located in central Israel had significantly higher risk of exposure, with 81% seroprevalence in comparison to 25%, 36.4%, 48.5 and 54.9% in the Golan Heights, the south, Palestine and the north, respectively. Horses located in central Israel were 12 times more likely to be exposed to EEV than in the Golan Heights (OR: 12.75, 95% CI: 5.2–30.9, *P* < 0.001). Seroprevalence in different breeds varied between 0 and 100%, although some breeds were represented only by a few horses (1–80 in each breed and 213 mixed breeds). When breeds were grouped (Arabians; Thoroughbreds and Warmbloods; Quarter horses, Paints and Appaloosas; Ponies; Gaited horses; Draft horses) it was found that gaited horses were 10 times more likely to be exposed to EEV than other breeds or mixed horses (OR: 9.84, 95% CI: 1.4–70.6). Geldings were more likely (64.6%) to be exposed than stallions (42.3%) or mares (50.8%) (OR: 1.84 95% CI: 1.22–2.78, *P* < 0.001, when stallions and mares were grouped together). Horses housed in stalls had significantly higher (*P* = 0.003) seroprevalence (70.3%) than horses in paddocks (53.1%) or pasture (49.6%). Horses in stalls were twice more likely to be exposed than in pasture (OR: 2.41, 95% CI: 1.41–4.11, *P* < 0.001). The mean age of the horses exposed to EEV was significantly higher than the mean age of the seronegative horses (11.4 ± 0.36 years and 8.8 ± 0.41 years, respectively). Also, an increase in seroprevalence was observed with increase in age categories (<3, 3–6, 6–9, 9–12, >12 years) with a significant linear association (*P* < 0.001) (Fig. 3).

All factors that were found significant in the univariable analysis were included in multivariable logistic regression. Only horses from Israel were included in this analysis since housing information was no available regarding the horses from Palestine. The farm (*P* < 0.001) and horses age (*P* = 0.003) were the only factors found significant in this analysis.

Associations between factors were assessed to control confounders. The farm was found to be associated with the geographical location, horse breed, horse sex and housing (all *P* values<0.001). To control the effect of the farm as a confounding factor, the data were analysed using generalized estimating equation, with the farm set as a random variable. The factors that were found significant in this model were the geographical area (the centre more prone to exposure, *P* < 0.001) and horse age (*P* = 0.003).

## Discussion

EEV in an important arbovirus that has been reported to be endemic in Israel since 2001 (Wescott *et al*. 2013). EEV is transmitted through the bites of certain *Culicoides* spp., and has relatively mild clinical manifestations in most cases. Monitoring EEV exposure may have a predictive value to assess the risk for outbreaks or introduction of other, more clinically and financially important arboviruses, such as AHSV, BTV and EHDV. In recent years, African horse sickness (AHS) was diagnosed in several African countries (Angola and Botswana in 2009, Eritrea in 2012, Mozambique and Swaziland in 2014, Senegal in 2015) and it is reported yearly in Ethiopia, Namibia and South Africa (Available at: http://www.oie.int/wahis_2/public/wahid.php/Diseaseinformation/Diseasetimelines, accessed 1 May 2016). There is a concern that AHSV, like EEV, might also enter the region, where it was diagnosed for the last time in 1944 (Available at: http://www.vetserv.moag.gov.il/Vet/all_Publications/dochot-shnatiim/default.htm, accessed 1 May 2016), or one of its neighbouring countries. A recent study of EEV prevalence in Israel found fluctuations in seroprevalence at different time points (65.1% in 2002, 36.5% in 2007, 64.5% in 2010 and 77.2% in 2011) and suggested that certain climatic conditions and herd immunity influenced the risk of exposure (Aharonson‐Raz *et al*. 2015). The current seroprevalence in Israel reported here was lower than reported in 2011 (*P* < 0.001), and was 58.2%. Studies from South Africa found variations in EEV annual incidence and circulating serotypes and demonstrated that pyrexia does not necessary correlate with infection (Howell *et al*. 2008; Grewar *et al*. 2015). Only one serotype of EEV had been isolated in Israel that was similar, but not identical to the South African EEV‐3 (Aharonson‐Raz *et al*. 2011), and the method used for detection of specific antibodies was based on this isolate (Aharonson‐Raz *et al*. 2014). It is possible that other EEV serotypes exist in the area, but since none was isolated it was impossible to construct a valid test for their detection.

EEV seroprevalence was found to increase with age. This correlated with the findings of the previous survey, at all time points (Aharonson‐Raz *et al*. 2015), and reflected the fact that the presence of antibodies indicates previous exposure to the virus, and not necessarily a recent one. It is probable that horses older than 7 years of age were exposed to the virus during the 2008 outbreak. However, we also found that even in horses <7 years of age, seropositivity increased with age, as might be expected as the timeframe for infection increased. A similar trend was also found in the seroprevalence of West Nile virus, another arbovirus prevalent in Israel and transmitted by mosquitoes (Aharonson‐Raz *et al*. 2014). Housing was also found to influence exposure to EEV. Horses housed in stalls were more likely to be seropositive than horses housed in paddocks or pasture. In the previous survey it was found that horses in stalls or paddocks were more likely to be seropositive than horses in pasture (Aharonson‐Raz *et al*. 2015). Since farm management correlated with geographical location, it is hard to distinguish between the two. Most horse pastures in Israel are located in the Golan Heights or in the north that where regions with lower EEV prevalence, whereas most horses in the central region, which is more densely populated, and had the highest EEV prevalence, are housed in stalls.

To better understand the epidemiology of EEV in the Middle East we compared the seroprevalence in Israel to Palestine and to Jordan. Due to technical limitations, samples in Palestine were not collected during the same time period as the samples from Israel, however, since prevalence in Israel was previously reported to be high (77%) (Aharonson‐Raz *et al*. 2015), we expect seasonal incidence to be low, furthermore, all horses that were sampled did not have febrile disease in the months before sampling and ephemeral outbreak was not reported in both Israel and Palestine during these months, therefore we assume that the several‐months difference between the sampling in Israel and in Palestine have no significant effect on the seroprevalence that was found. Seroprevalence in Palestine was 48.5% and did not differ significantly from Israel (58.2%). The slightly lower prevalence may be explained by the fact that in Palestine most samples were collected from locations within the Jerusalem mountain region, where the higher altitude may result in lower abundance of *Culicoides* biting midges. Lower exposure of horses to *Culicoides* midges at higher altitudes was suggested previously as an explanation for the finding that horses from farms more than 800 m above sea level did not suffer from *Culicoides* hypersensitivity as opposed to horses located in farms less than 300 m above sea level in Israel (Steinman *et al*., 2003). The prevalence in the Golan Heights area in Israel was also lower than in other regions (25%), maybe for the same reason. EEV seroprevalence in Jordan was significantly lower than in Israel and Palestine and only two of 100 horses were seropositive. The farms sampled in Jordan held sport horses which were maintained in very restrict conditions, and were all located in the same geographical province (around Amman), and therefore, may not represent the Jordanian horse population correctly. Furthermore, due to technical limitations, horses from a small number of farms were sampled in both Palestine and in Jordan, therefore, the horses sampled did not represent the equine population in these regions, and results only suggest exposure to this virus. The ability of pathogens to invade new regions is dependent on the frequency of their introduction and the existence of adequate environmental conditions to support their vector population in the invaded area. Data regarding the prevalence of different *Culicoides* species in Jordan are limited but a Simbu serogroup virus closely related to Aino virus, which is transmitted by *Culicoides* midges, was recently reported in dairy farms located in northern Jordan (Abutarbush *et al*. 2015), indicating that *Culicoides* midges are found in that area. A more extensive survey should be constructed to better reflect the equine population in Jordan.

In conclusion, EEV seroprevalence in Israel in 2015 was significantly lower than in 2011. The seroprevalence in Israel and Palestine did not differ statistically. The seroprevalence in Jordan was significantly lower than in Israel and Palestine, but may not represent the entire horse population in the country. Seroprevalence was found to increase with age. Different management practices at different farms and lower abundance of *Culicoides* in higher altitude may explain some of the differences in prevalence in different farms and areas. This is the first comparative report of EEV in the Middle East that may shed more light on EEV dynamics in this important geographical junction.

## Source of funding

This research was supported financially by grant 2014.52146 funded by the Netherlands Ministry of Foreign Affairs, The Hague, Netherlands.

## Conflict of interest

The authors declare that they have no conflicts of interest.

## Contributions

The authors have no additional contributions to declare.
